# A Narrative Review of Urbanization and Mental Health: An Indian Perspective

**DOI:** 10.7759/cureus.55381

**Published:** 2024-03-02

**Authors:** Ankita Hepat, Dipali Khode, Swarupa Chakole

**Affiliations:** 1 Public Health, Jawaharlal Nehru Medical College, Datta Meghe Institute of Higher Education & Research, Wardha, IND; 2 Epidemiology and Public Health, Jawaharlal Nehru Medical College, Datta Meghe Institute of Higher Education & Research, Wardha, IND; 3 Community Medicine, Jawaharlal Nehru Medical College, Datta Meghe Institute of Higher Education & Research, Wardha, IND

**Keywords:** urbanization programs, urban health hazard, impact on women's mental health, mental health, urbanization

## Abstract

Urbanization is a phenomenon that shows the expansion of urban areas in conjunction with industrial and economic progress. Rapid world urbanization is caused by the swift rise in urban residents as a proportion of the population. Efforts to examine the quality of urban life and urbanization as distinct risk factors for mental illness within specific age groups have been made. However, the issue remains contentious and largely unresolved. Urban mental health issues, such as substance abuse, fear of crime, poverty, and ethnicity, are associated with factors like depression, aggression, fear, sadness, and personality disorders. The entire gamut of the population, particularly adult males and females, is affected by these impacts of urbanization. The size of the population increases the prevalence of the preview study. India is expected to concentrate highly on the whole urban population, but this might inadvertently cause harm to urban residents. This review discusses the impact of urbanization on mental health and well-being. We search via PubMed (Medline), Google Scholar, and databases like WHO. The language of the study is English, and other language articles are excluded. The mental health challenges associated with urbanization impact the entire population, with a notable emphasis on adult males and females. They are raising awareness about various urban programs designed for urban populations. It will function as a coordinator of change in a rapidly modified Indian society to spread awareness about mental illnesses throughout all segments of society.

## Introduction and background

The concept of mental health is defined by the WHO as a condition of well-being in which individuals acknowledge their capacity, effectively manage their typical life stresses, participate in meaningful and productive activities, and contribute to the well-being of their communities [1]. Urbanization is a complex process characterized by the expansion of cities resulting from industrialization and economic advancement. This phenomenon brings about distinctive urban-specific transformations in specialization, the division of labor, and people’s behavior [2]. The community composition of a state and the global, national, and regional economies are significantly influenced by urbanization. While it is acknowledged that the fulfillment of expectations regarding education, services, employment, cultural enrichment, and improved health may not be consistent across the board, urban areas are generally perceived, in theory and popular belief, as providing better opportunities [3]. One of the crucial world health problems of the 21st century is the rapid increase in urban individuals worldwide. Projections from the United Nations Population Division indicate that by 2030, a larger proportion of individuals in the developing world will be living in cities as opposed to rural areas. Urbanization is likely to encompass two-thirds of its population by 2050. This trend also influences the scenario in India. The UN World Urbanization Prospects 2008 estimates that 28% of India’s population now resides in cities, and that number will rise to 41% by 2020 [4].

Rapid and often unplanned urban growth poses risks such as poverty, unemployment, environmental degradation, and the strain of population demands surpassing service capacity. These situations place individuals’ health at risk [5]. The health risks associated with urban health hazards include factors such as air pollution, inadequate or contaminated drinking water, overcrowding, poor housing, and a lack of sanitation and solid waste disposal services. Additionally, urban areas may face challenges such as industrial waste, a high incidence of increased motor vehicle traffic, vector-borne diseases, and stress related to unemployment and poverty [5]. As individuals migrate and adjust to urban living, the practical aspects of the urban lifestyle exert a considerable effect on the lives of various people, despite their pursuit of these opportunities. The vulnerable sections, like pregnant women, children under five years, and geriatric people, suffer the most [3]. This aims to illuminate the urbanization process, its challenges, and the implications it holds for the mental health and well-being of urban dwellers [6].

In an urban setting, a range of diseases and deviancies may emerge. These include depression, sociopathy, psychoses, substance abuse, alcoholism, delinquency, crime, and vandalism, as well as family disintegration and separation [7]. Neglect persists in addressing mental health concerning the measurement of the problem’s scope and causes, as well as the testing of interventions to alleviate suffering [8]. To create focused intervention and prevention programs that encompass both urban planning and population psychosocial initiatives, it is insufficient to view the urban city as a singular overarching risk factor or to solely focus on remote environmental factors [9].

The World Urbanization Prospects Report indicates that the repercussions of the rapid pace of urbanization, leading to sustainable development challenges, will be more perceived in urban areas, especially in lower-middle-income countries such as India [10]. Social and economic advancement are facilitated by well-managed urbanization. Continuous urbanization is anticipated to result in the alleviation of poverty and hunger, along with an increase in prosperity. In the fiscal year 2009-2010, urban areas contributed 63% to India’s gross domestic product (GDP), and it is expected that this proportion will escalate to 75% by the year 2030 [10].

The United Nations reported in 2016 that one-third of the global community will be residing in cities by 2030, indicating that the lifestyles of most people will soon be influenced by urbanization [11]. Urbanization is an essential component of economic growth. In many countries, including India, towns and cities play a significant role in contributing to the country’s economy. Urban areas, which make up less than one-third of India’s population, account for more than two-thirds of the GDP and 90% of government revenue [12]. Urban areas in India are inhabited by more than 35% of the individuals, approximately equivalent to 48 crore people, with an annual increase of 2.34%. It is anticipated that by 2030, more than half of India’s population will live in cities. Public utilities include housing, transportation, electricity, water, sanitary conditions, health, and education [13]. In 1960, 34% of the total global population constituted the urban population. However, by 2014, the proportion of individuals living in cities had risen to 54% of the total and was still growing. It is expected that by 2050, 66% of the population will live in urban areas [14].

## Review

Methodology

**Figure 1:**
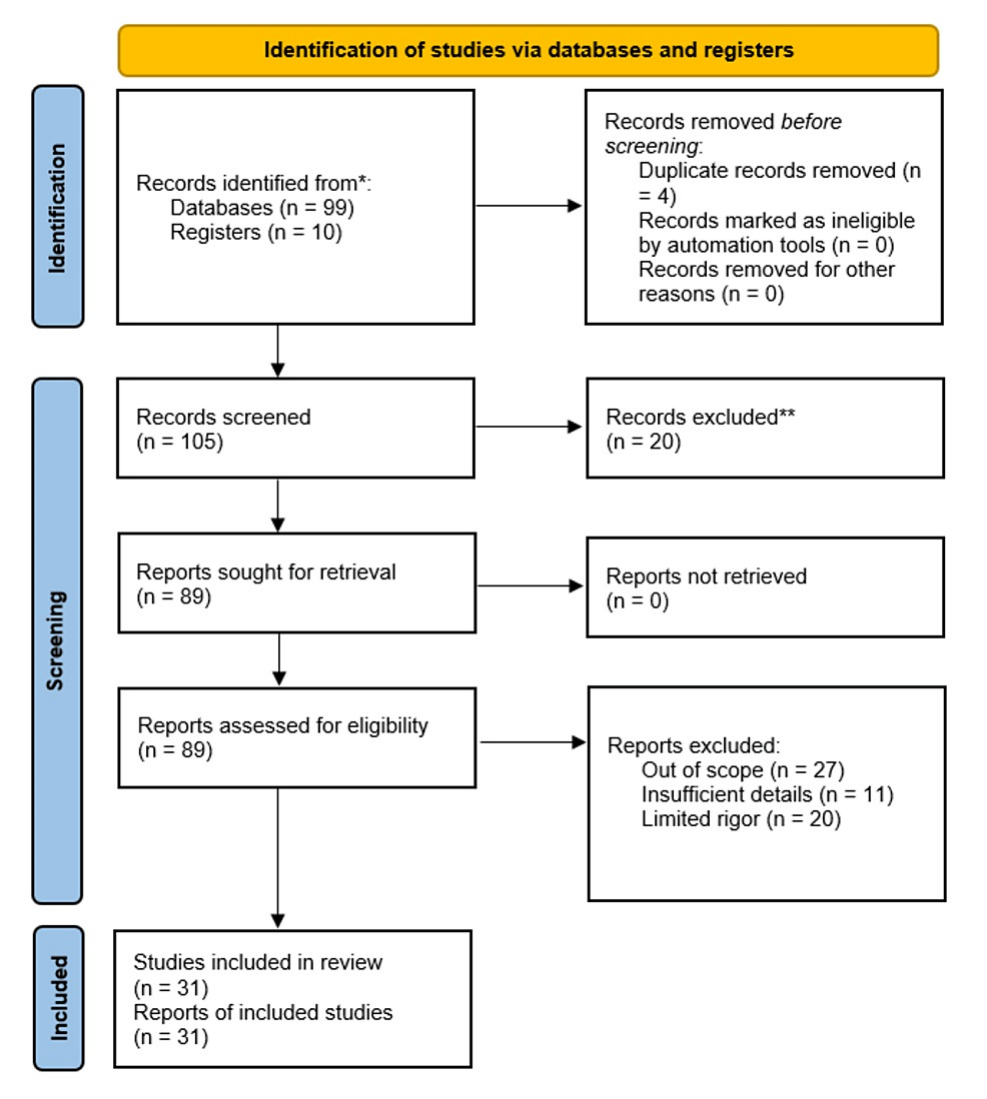
PRISMA methodology for literature search Adapted from the PRISMA guidelines PRISMA, Preferred Reporting Items for Systematic Reviews and Meta-Analyses

This review discusses the impact of urbanization on mental health and well-being. We conduct searches through PubMed (Medline), Google Scholar, and databases such as WHO for our research. The PubMed search strategy was customized for specific databases, encompassing the following manner: (((impact[Title/Abstract]) OR (urbanization{MeSH Terms])) AND (urban area[MeSH Terms])) AND (mental health[MeSH Terms])) AND (well-being[MeSH Terms]). Moreover, we thoroughly examined the reference lists of potentially relevant studies to identify additional research. The study is conducted in English, and other language articles are excluded. Articles ranging from 2000 to 2023 were used. For more detail, we used various keywords like “impact of mental health,” “urbanization,” “well-being,” “mental health,” “urbanization programs,” and “impact of women’s mental health.” Additional filters, such as free full text, are applied to all research (Figure 1).

Table 1 shows the summary of the included studies.

**Table 1:** List of included studies in the review

Sr. no	Author	Year	Type of study	Finding
1	Turan and Beşirli [2]	2008	Review article	Negatively impacting mental health, it becomes a significant process that should not be disregarded.
2	Hiremath [3]	2022	Review article	There is a necessity to raise awareness about mental illnesses throughout all sectors of the community.
3	Srivastava [4]	2009	Review article	Awareness of its impact on health, especially mental health, will serve as a catalyst for change in the growing Indian economy.
4	Trivedi et al. [5]	2008	Review article	Urbanization has an impact on the entire spectrum of individuals, particularly vulnerable groups such as the elderly, children, adolescents, and women.
5	Halbreich [6]	2023	Review article	Government policies and funding should be reprioritized.
6	Fauzie [7]	2015	Review article	A study on rural-urban migration revealed that older individuals were more likely to report depressive symptoms.
7	Ludermir and Harpham [8]	1998	Review article	This study is criticized for excessively focusing on single risk factors instead of considering the community and structural context.
8	Xu et al. [9]	2023	Original article	Our findings suggest that varying environmental profiles of urban living may have effects, particularly in psychiatric symptom groups between unique neurobiological pathways.
9	Abhishek et al. [10]	2017	Review article	This study highlights a growing need for improved healthcare policies and infrastructure development to sustainably support the growth of Indian urban agglomerations.
10	Bai et al. [15]	2012	Journals and books	We conclude by recognizing the key elements necessary for the success of the new science initiative.
11	Mishra [16]	2021	Journal article	The current emphasis is on the development of intervention programs to enhance the mental health of women.
12	Jaysawal and Saha [17]	2014	Journal article	Through an analysis of its multidimensional impact, this study examines the effects of fast-growing urbanization on Indian society.
13	Moore et al. [18]	2003	Review article	There is a need for systematic and useful disaggregated urban health statistics.
14	Kjellstrom et al. [19]	2007	Journal article	Preparing the living environment for the WHO Commission on Social Determinants of Health, the Knowledge Network on Urban Settings aims to improve it.
15	Kar and Somani [20]	2015	Review article	Increasing general awareness among society and mental health professionals about this pressing issue will greatly contribute to finding a long-lasting solution to the problem.
16	Malhotra and Shah [21]	2015	Review article	The findings of this study suggest that coordinated efforts at social, political, economic, and legal levels can bring about change in the lives of Indian women and contribute to the improvement of their mental health.

Discussion

**Figure 2:**
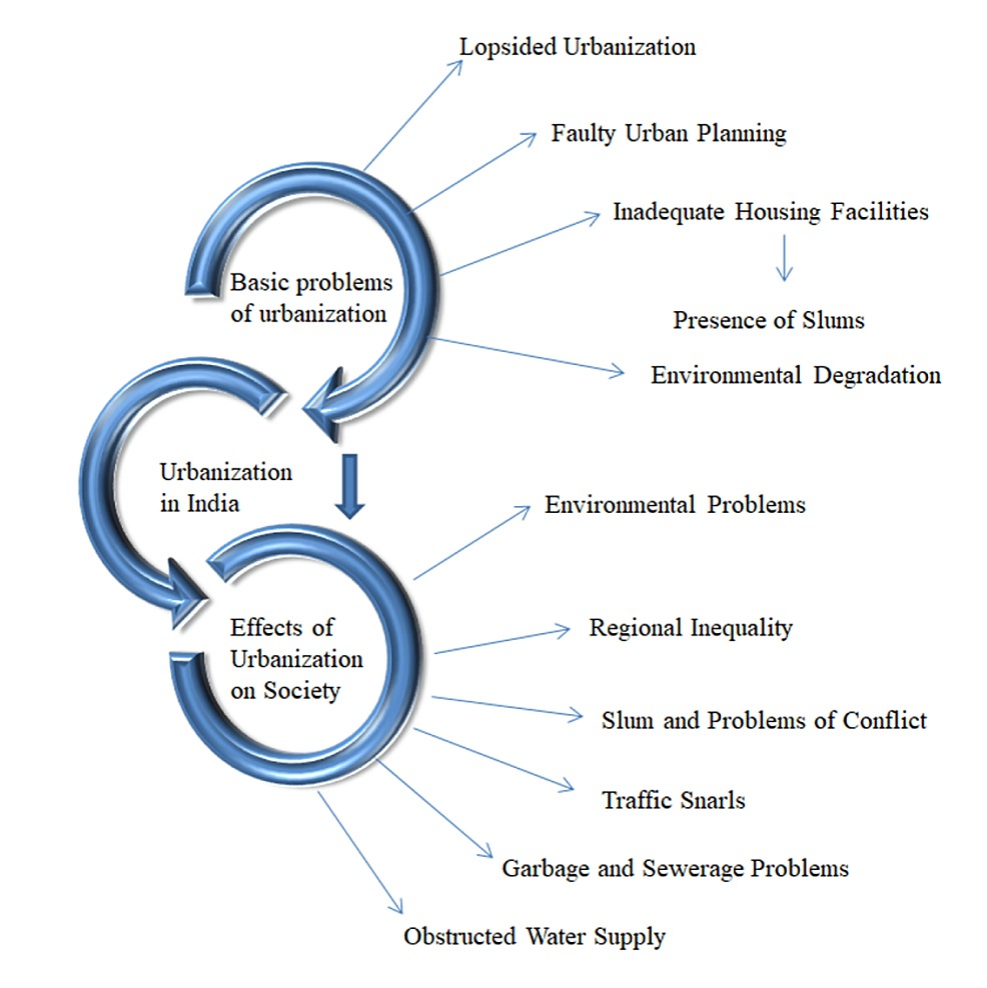
The interface between issues and effects of urbanization in India Image credit: Ankita Hepat

The 2018 Revision of World Urbanization Prospects, developed by the United Nations Population Division and the Department of Economic and Social Affairs, projects a significant increase in the global urban population, with a predominant concentration expected in India [3]. As found in the previous study, in an urban setting, a higher income leads to a more comfortable lifestyle, which ultimately results in a reduced level of anxiety [16]. This model shows the cases of interconnectedness between issues and the effects of urbanization in India. It highlights the impact of urbanization on Indian society by examining it from various perspectives. It aims to establish a connection between the negative aspects of urbanization and their impact on societal progress [17]. Figure 2 depicts the interface between the issues and effects of urbanization in India.

Urban Health Hazards

The environment has been severely impacted by urbanization. Other significant trends, such as megacities and industrialization [3], various urban health disorders, and related risks, arise from inadequate housing on marginal land, overcrowding, high air and water pollution, insufficient solid waste collection, insufficient sanitation services, as well as motor vehicle traffic and associated injuries. These issues are related to the rapid expansion of urban centers [18], which negatively impacts the growth of children and even has effects on the brain [3]. In the developing world, morbidity and mortality are significantly caused by air pollution, impacting urban society. Unhealthy living and working conditions can exacerbate noncommunicable diseases like asthma, cancer, cardiac disease, and diabetes mellitus. Contributing factors encompass inadequate green spaces, various types of pollution like water, noise, and soil contamination, urban heat islands, and a lack of places that encourage cycling, walking, and active lifestyles. Obesity and physical inactivity in urban areas with insufficient transit and walking/cycling infrastructure are associated with diabetes. Urbanization is also related to increased rates of anxiety, depression, and other mental health problems [22]. Nearly half of the urban populace grapples with these illnesses due to limited access to enhanced water, sanitation, and exposure to air pollution [19].

Impact of Urbanization on Mental Health

A WHO report has outlined that mental illness makes up approximately 12% of the global burden of disease. By the year 2020, it was estimated that these conditions would account for almost 15% of disability-adjusted life years lost to illness. Incidentally, young adults, deemed the most productive age group of the population, bear the maximum burden of mental disorders [4]. Mental health disorders represent a characteristic urban phenomenon, as features like life instability, insecurity, and mental isolation prevail in urban communities. Mental disorders arise from a lack of emotional satisfaction in an individual [3]. Meta-analytic studies have frequently observed the relationship between living in urban environments and increased psychological distress, along with the risk of mental disorders including anxiety, depression, psychosis, crime, alcoholism, and other substance use disorders [23]. Several studies comparing the populations of rural and urban areas have shown that mental disorder rates have increased in cities [20]. Indian epidemiological research on mental health disorders indicates that the frequency of mental illness ranges from 48.9% in rural areas to 80.6% in urban areas [20].

Impact on Women’s Mental Health

The WHO analysis in 2005 documented a close association between women’s mental health and the experience of violence [4]. Gender discrimination, malnutrition, overwork, and incidents of domestic and sexual violence contribute to the challenges associated with women’s mental disorders [7]. According to epidemiological research conducted in different parts of India, 64.8 out of 1,000 women have psychological disorders [3]. Out of 2,212 women, 23% showed elevated levels of CRP, and 21% were identified as overweight or obese. Among the women surveyed, one-third were situated in highly built-up areas, and 29% resided in urban settings [24]. In contrast, a lower income results in poverty and an escalation in the working hours of women within the household. Ultimately, the disturbance of family peace and happiness during hours of grief and tension renders women more susceptible to psychological traumas [16]. Particularly vulnerable, women often bear a disproportionate burden associated with the changes brought by urbanization. In a rural setting, women would primarily work within their homes. However, the predominantly nuclear setup in cities, associated with economic pressures, compels women to venture outside [5]. Despite having a more substantial role in an urban structure, women still lack the corresponding increase in hierarchy in the community that should rightfully accompany the rising demands placed on them [5].

The current population-based study conducted in India revealed that almost half of the women reported encountering physical violence, with a high prevalence of domestic violence observed in urban areas [2,23]. Depression, organic brain syndromes, and dementias are identified as the primary mental health issues among the elderly. The majority are women, and between 16% and 50% of cases involve violence against women [21]. Women, being particularly vulnerable, often bear a disproportionate burden associated with the changes brought by urbanization [4].

Psychological Impact

The WHO researchers investigated the occurrence and correlates of mental illness through cross-national comparisons in their study. A persistent pattern shows that mental illnesses are more common in urban areas than in rural places [5]. Mental and neurological disorders in the elderly, such as Alzheimer’s disease, other forms of dementia, and depression, play a substantial role in contributing to the prevalence of noncommunicable diseases [1]. Psychological disorders are thought to be caused by the model of cultural transformation, specifically the shift from rural to modern society. Most dementia sufferers are found in emerging nations; from 60% in 2001 to 71% by 2040, this proportion is expected to rise. The rates of growth are not consistent; while numbers are expected to double in developed countries between 2001 and 2040, the projection indicates an increase of over 300% in India. A higher prevalence of mental disorders was observed in cities, specifically 80.6%, in contrast to the 48.9% recorded in rural areas. Depression and neurotic disorders compose the largest number of mental disorders [4]. The conditions have a negative impact on the social, economic, and psychological traits of individuals and communities residing in cities. One can assert that the association between the urbanization process and mental health is influenced significantly by social, economic, and political factors [2]. Chronic problems, including poor, congested physical settings, high rates of accidents and violence, precarious housing, and unstable tenancy, have all been connected to depression. In developing countries, the primary cause of disease burden is projected to be major depression [5].

Public Health Problems in Urban

**Figure 3:**
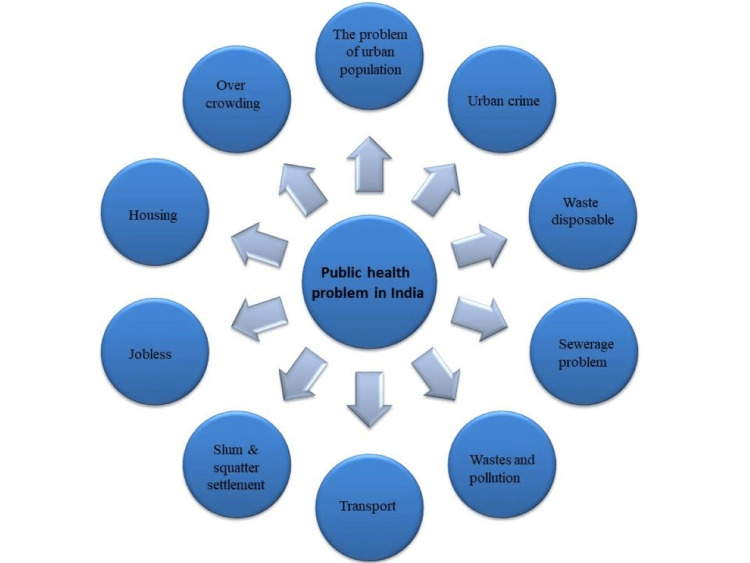
Public health problems in urban areas Image credit: Ankita Hepat

As a city expands, its land use pattern changes, leading to both horizontal and vertical growth. The horizontal expansion often engulfs nearby fringe villages and converts agricultural lands, resulting in a decrease in water level. This situation increases the risk of drinking water contamination due to pipe leakage. Additionally, the scarcity of land in growing urban centers causes a rise in land values. Consequently, there is a proliferation of apartment buildings, often approved without adequate consideration of sewage facilities in busy centers. Hence, there is a mushrooming increase in apartments, and in busy centers, the apartments are permitted without scrutinizing the way sewage infrastructure provisions are made [17]. The public health problems in urban India may be seen on various lines. The following components include the problems of the urban population, urban crime, waste disposal, sewerage problems, waste and pollution, transport, slum and squatter settlement, joblessness, housing and overcrowding, etc. Figure 3 depicts public health problems in urban areas [17].

Programs in India Related to Urbanization

India’s urban population is concentrated in about 30% of places. Because of the country’s fast urbanization, which is closely linked to overall economic growth, cities are now dealing with serious socioeconomic issues. These challenges include issues like unemployment and overburdened existing infrastructure in areas such as housing, sanitation, transportation, health, education, utilities, etc. The Ministry of Housing and Urban Development has rapidly founded new projects, spreading awareness about urbanization programs in India, and renovating current ones targeted at solving these particular issues to improve people’s quality of life, particularly for the urban poor [25]. Table 2 depicts urbanization programs in India.

**Table 2:** Urbanization programs in India

Sr. no	Name of programs	Years	Launch under ministry	Aims	Objective
1	Smart Cities Mission	June 25, 2015; officially launched on June 25, 2016	Ministry of Housing and Urban Affairs	The aim is to construct 100 smart cities throughout India, emphasizing planned urbanization and sustainable development to serve as a supportive framework for neighboring cities.	Program goals include modernizing communities through the use of “smart” solutions, clean and sustainable environments, essential infrastructure, and a respectable standard of living for residents [25,26].
2	Pradhan Mantri Awas Yojana (Urban) - Housing for All Mission	June 25, 2015	Ministry of Housing and Urban Poverty Alleviation	The goal is to house every urban citizen by 2022.	Through the use of land as a resource, private developers assist in the rehabilitation of slum dwellers [26,27].
3	Swachh Bharat Mission - Urban (SBM-U)	October 2, 2014	The Prime Ministry of India launched the Swachh Bharat Mission.	The aim is to enhance infrastructure and supply additional amenities to urban areas.	The goal of attaining universal coverage of sanitation in urban areas is complemented by a financial allotment of Rs 41,765 crore for the 2018-2019 fiscal year [25,26].
4	Atal Mission for Rejuvenation and Urban Transformation (AMRUT)	Launched in 2015	The government is transforming urban living conditions through infrastructure upgradation.	The aim is to revitalize 500 cities and towns into highly functional urban living environments within a span of five years.	Ensure that each household has access to a tap with a reliable water supply and is connected to a sewerage system [26,28].
5	Jawaharlal Nehru National Urban Renewal Mission (JNNURM)	Launched in 2005	The Government of India under the Ministry of Urban Development	The aim is to promote, enhance, and expedite the planned development of designated cities.	Focused attention on the integrated infrastructure service development in the urban areas under the Mission [27,29].
6	Heritage City Development and Augmentation Yojana (HRIDAY)	January 21, 2015	The Government of India’s central sector schedule	The goal is to inclusively incorporate urban planning, economic development, and heritage preservation.	Preserving the city’s historic character is the goal [30].
7	Deendayal Antyodaya Yojana-National Urban Livelihoods Mission (DAY-NULM)	September 24, 2013	Ministry of Housing and Urban Poverty Alleviation.	The goal is to improve sustainable livelihood prospects through skill development, lifting the urban poor.	Making India’s goal of developing skilled labor is crucial for improving socioeconomic conditions [25,31].

## Conclusions

Impacts related to mental health behavior were brought by urbanization. There is compelling proof indicating that urban individuals were significantly more susceptible to reporting mental illness compared to their nonurban counterparts. Numerous factors, such as urban health hazards and causes, are associated with air pollution, water pollution, poor sanitation, depression, somatic symptoms, social maladaptation, and psychological disorders. The total spectrum of the population, particularly the vulnerable sections such as the elderly, children under five years, and women, is affected by urbanization. There is a higher prevalence of depression among women, such as housewives, who are suffering from stress and anxiety. In urban areas, people migrate from rural areas to urban cities for the betterment of living standards and also to improve the quality of life and take advantage of more opportunities that will be easily available and accessible to them. So the government overcame this situation by launching some programs and filling the gap. The government appears to provide facilities for those they choose to achieve an equitable distribution.
